# Prognostic Factors after Surgery for Salivary Gland Cancer; What Is New, and What Is Next?

**DOI:** 10.3390/cancers14092130

**Published:** 2022-04-24

**Authors:** Angelo Camaioni, Pietro De Luca, Francesco Antonio Salzano

**Affiliations:** 1 Otolaryngology Department, San Giovanni-Addolorata Hospital, 00184 Rome, Italy; acamaioni@hsangiovanni.roma.it; 2 Department of Medicine, Surgery and Dentistry, University of Salerno, 84084 Salerno, Italy; frsalzano@unisa.it

Salivary gland cancers account approximately for 7% of all head and neck tumors [1]; these neoplasms can arise from major salivary glands (the parotid gland, submandibular gland, and sublingual gland) or from minor salivary glands in the upper aerodigestive tract [2]. Despite showing a low incidence as compared to other head and neck cancers, data suggest that their incidences have increased during the last twenty years. In addition, salivary gland cancer-related deaths have not significantly decreased. 

Radical or partial resection based on tumor stage and histopathological diagnosis remains the gold standard, which can be followed by adjuvant radiotherapy (RT) treatment, depending on the presence of some prognostic factors.

In the last decade, the introduction of new classifications in parotid surgery, such as the classification of branching pattern of facial nerve during parotidectomy proposed by Alomar and the European Salivary Gland Society’s classification of parotidectomies [3,4], have gained popularity; moreover, to reach the best cosmetic result with minimal access, new surgical approaches, such as the endoscopic retroauricular approach for benign and malignant parotid tumors [5,6,7] and the robotic surgery for parotid and submandibular cancers [8,9,10], have attracted the interest of head and neck surgeons.

In the era of precision medicine, the identification of molecular biomarkers for cancer detection is one of the greatest challenges for all clinicians, including ENT specialists, aiming to identify new prognostic factors of poorer and better outcomes.

In this light, liquid biopsy is a new, safe, and minimally invasive diagnostic tool that individuates circulating tumor cells (CTCs) and circulating free DNA (cfDNA) to identify candidate therapeutic targets and to early detect tumor recurrence [11,12]. 

Metcalf et al. reported the first application of liquid biopsy to adenoid cystic carcinoma (ACC), showing some potential to detect clinically actionable mutations, to define the tumor profile, and to early detect tumor recurrence [13]; additionally, a recent review from Zhang et al. explored the potential role of extracellular vesicles (EVs) and exosomes in monitoring salivary gland cancers, confirming the potentiality of the technique despite the majority of the studies having been conducted in vitro [14].

Emerging clinicopathological predictors of survival have been recently identified for acinic cell carcinoma (AciCC), mucoepidermoid carcinoma (MEC), small cell neuroendocrine carcinoma “Merkel type” (SNECM), salivary duct carcinoma (SDC), and other major salivary neoplasms [15,16,17,18,19,20,21,22,23].

The aim of this Special Issue is to stimulate discussion about news in major salivary gland surgery, trying to provide evidence-based data focused on new prognostic factors in major salivary gland cancers, especially in parotid oncological surgery.
